# Protective effect of uridine on atrial fibrillation: a Mendelian randomisation study

**DOI:** 10.1038/s41598-023-47025-8

**Published:** 2023-11-10

**Authors:** Xintian Xu, Xiaoyu Zhang, Shiyao Cheng, Qinglang Li, Cai Chen, Mao Ouyang

**Affiliations:** 1 Department of Cardiology, The Sixth Affiliated Hospital, Sun Yat-Sen University, 26 Yuancun Erheng Road, Guangzhou, 510655 Guangdong People’s Republic of China; 2 Biomedical Innovation Center, The Sixth Affiliated Hospital, Sun Yat-Sen University, Guangzhou, People’s Republic of China

**Keywords:** Cardiology, Diseases, Endocrinology, Medical research, Risk factors

## Abstract

Uridine, a pyrimidine nucleoside, is crucial in the synthesis of metabolites. According to observational studies, a higher plasma uridine level is associated with a lower risk of atrial fibrillation (AF). However, the casual relationship between uridine and AF is still unknown. In this study, we used the Mendelian randomisation (MR) approach to explore causality. Three genetic variants associated with uridine were identified from the Metabolomics GWAS server (7824 participants); summary-level datasets associated with AF were acquired from a genome-wide association study (GWAS) meta-analysis with 1,030,836 European participants (60,620 AF cases). We duplicated the MR analyses using datasets from AF HRC studies and the FinnGen Consortium, and then conducted a meta-analysis which combined the main results. The risk of AF was significantly associated with the genetically determined plasma uridine level (odds ratio [OR] 0.27; 95% confidence interval [CI] 0.16, 0.47; *p* = 2.39 × 10^–6^). The association remained consistent in the meta-analysis of the various datasets (OR 0.27; 95% CI 0.17, 0.42; *p* = 1.34 × 10^–8^). In conclusion, the plasma uridine level is inversely associated with the risk of AF. Raising the plasma uridine level may have prophylactic potential against AF.

## Introduction

Atrial fibrillation (AF), which is one of the most prevalent arrhythmias worldwide, increase the risk of stroke and embolism. The morbidity of AF is rising, due in part to the aging of populations worldwide^1^. According to data from the Framingham Heart Study, AF morbidity rates have increased threefold in the past 50 years^2^. For all that, a set of risk factors include high systolic blood pressure, body mass index, diabetes, and the like, have been proven to this day. The identification of protective factors for AF may facilitate the development of effective preventive and therapeutic measures.

Uridine, a pyrimidine nucleoside and regeneration-related metabolite, rejuvenates aged stem cells and regenerates tissues in vivo^3^. As a precursor of uridine triphosphate, uridine activates the synthesis of glycogen, participates in the synthesis of various other metabolites, and exerts positive effects on improving cardiac function^4^. Ion channel malfunction and aberrant Ca^2+^ handling are implicated in the pathogenesis of AF^5^. In experimental animals, the uridine derivative uridine-5ʹ-diphosphate reactivated the mitochondrial ATP-dependent potassium channel in the mitochondria of rat heart at 30 μM, thus ameliorating rhythm disorders^6^. Also, uridine promoted maintenance of mitochondrial homeostasis, thereby enhancing Ca^2+^ homeostasis in the myocardium^4^. Observational studies of the relationship between uridine and AF yielded discrepant results. No study has assessed the causal association between the plasma uridine level and AF, which might be clarified by large-scale randomised controlled trials (RCTs), however such trails are costly.

Mendelian randomisation (MR) is an epidemiological method that can reveal the causal association between exposure and outcome variables robustly^7^. MR is based on simulating an RCT with random assignment of variants during gametogenesis, and investigating causal associations taking account of minor residual confounders and reverse causality^8,9^. Specifically, single nucleotide polymorphisms (SNPs) are identified as unconfounded instrumental variables (IVs) in an MR study to proxy the phenotypes of interest^7^. The exposure and outcome datasets of a two-sample MR analysis should be obtained from two independent data sources to enhance reliability and validity^9^. Hence, based on large genome-wide association study (GWAS) datasets, we first explored the protective effect of uridine on AF using a two-sample MR approach.

## Materials and methods

### Study design

**Figure 1:**
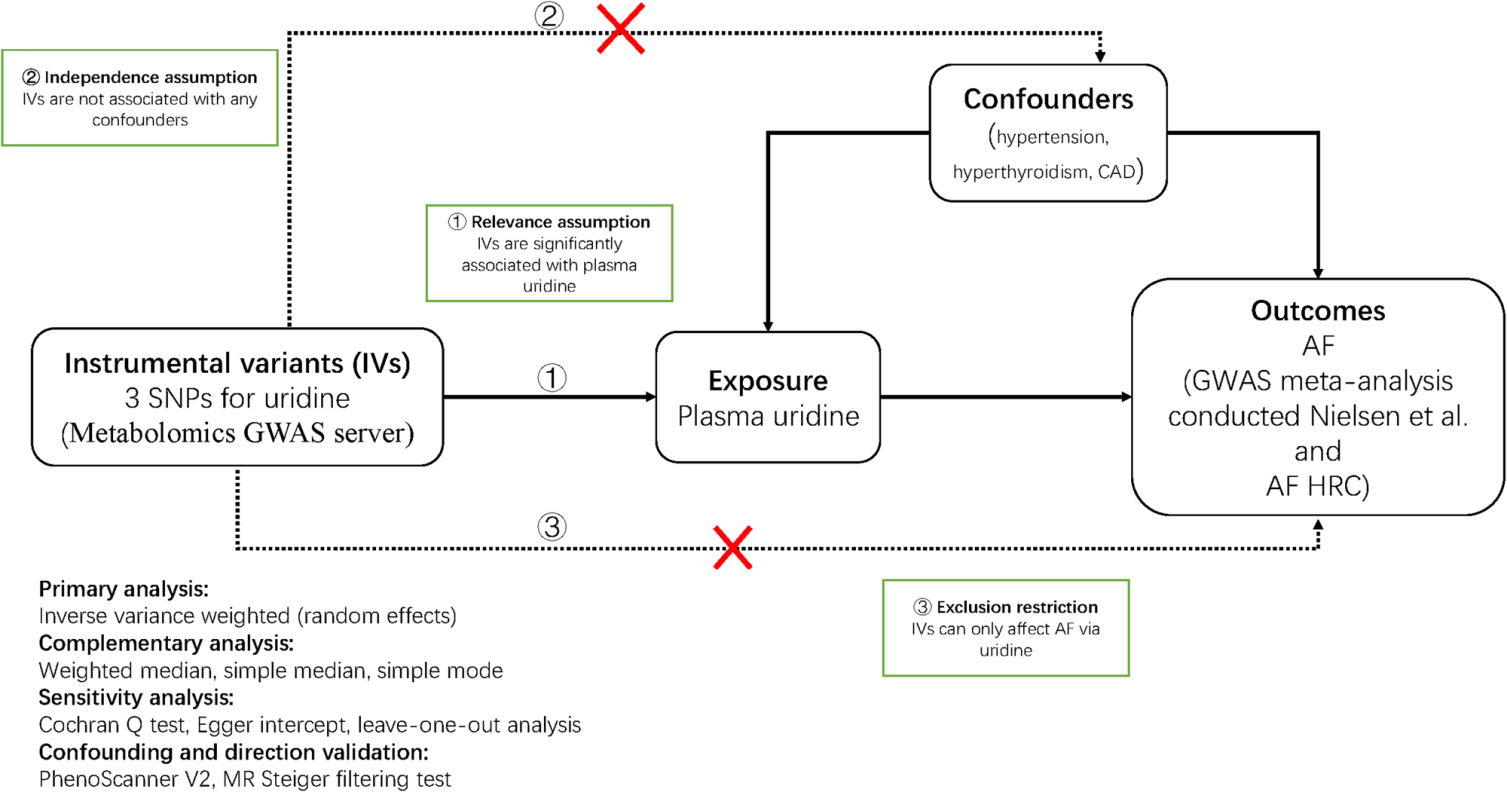
Mendelian randomisation framework diagram. *SNPs* single-nucleotide polymorphisms, *AF* atrial fibrillation, *CAD* coronary artery disease, *AF HRC* Atrial Fibrillation Haplotype Reference Consortium, *GWAS* genome-wide association study.

The causal relationship between uridine and the risk of AF was systematically investigated in a two-sample MR study. We selected SNPs as the IVs to proxy plasma uridine from a GWAS meta-analysis^10^, and confirmed the associations between IVs and AF in another large GWAS dataset^11^. There are three key assumptions^12^ that a convincing MR study must meet (Fig. 1). First, according to the relevance assumption, IVs should be significantly associated with plasma uridine; second, in light of the independence assumption, IVs should not be associated with confounders such as blood pressure, hyperthyroidism, or diabetes; third, in terms of the exclusion restriction, IVs should exert effects on the dependent variable (i.e. AF) only via (in this case) uridine. This study complied with The Strengthening the Reporting of Observational Studies in Epidemiology Using Mendelian Randomisation (STROBE-MR) standard (Table S1)^13^.

#### Ethic statement

All of the summary-level data used in this study are publicly available. Details regarding ethics approval and informed consent for each GWAS study included in this investigation can be found in the original publications.

### Data source and SNP selection

Uridine-associated SNPs were identified from the Metabolomics GWAS server^10^, which included the Cooperative Health Research in the Region of Augsburg (KORA) study (1768 participants of European ancestry)^14^ and UK Adult Twin Registry (TwinsUK) cohort (6056 participants of European ancestry)^15^. Summary-level GWAS data for the association analysis between the uridine-associated genetic variants and AF were obtained from two large GWAS meta-analyses: (1) a primary meta-analysis of the Nord-Trøndelag Health Study (HUNT), deCODE, Michigan Genomics Initiative (MGI), DiscovEHR, UK Biobank, and AFGen Consortium, which encompassed 1,030,836 individuals of European descent (60,620 AF cases; and 970,216 controls)^11^ and (2) the Atrial Fibrillation Haplotype Reference Consortium (AF HRC), which comprised more than 50 studies (Table S2) including 588,190 individuals (65,446 AF cases; and 522,744 controls)^16^, mainly (91%) of European ancestry (Table 1). In the first GWAS dataset, AF was diagnosed according to the International Classification of Diseases (ICD)-9 or ICD-10^11^. In addition, we used the outcome datasets from the FinnGen Consortia^17^ to confirm the results of the analyses (Table 1). The sixth wave of GWAS data on AF was used, which comprised 28,670 cases (defined by ICD-8 code 42792, ICD-9 code 4273, and ICD-10 code I48) and 135,821 controls.

**Table 1:** Studies and datasets used in the analysis.

Data source	Phenotype	Sample size	Cases	Population	Adjustment
Metabolomics GWAS server	uridine	7824	–	European	Age, gender, and body mass index
GWAS meta-analysis (Nielsen et al*.*)	AF	1,030,836	60,620	European	Age, sex, and principal components 1–4
AF HRC	AF	588,190	65,446	91% European	Age, sex, and principal components 1–10
FinnGen	AF	164,491	28,670	European	Age, sex, and up to 20 genetic principal components

*AF* atrial fibrillation, *AF HRC* Atrial Fibrillation Haplotype Reference Consortium.

The Metabolomics GWAS Server provided 129 SNPs significantly associated with uridine at *p* < 1 × 10^–5^^10^. To identify eligible genetic variants associated with uridine, a genome-wide significance threshold of *p* < 5 × 10^–8^ was set, excluding 118 SNPs that violated the relevance assumption. Next, to avoid bias from linkage disequilibrium (LD), we used the method of the 1000 Genomes European Project^18^ to perform clumping (r^2^, 0.001; clumping window, 10,000 kb)^19^. Then, we used PhenoScanner V2^20^ to identify potential confounders (i.e. hypertension, hyperthyroidism, and coronary artery disease) significantly associated (p < 5 × 10^−8^) with the SNPs. To test the exclusion restriction assumption, SNPs with reverse causality were detected using the MR Steiger filtering method^21^ (Table S3). Finally, three SNPs associated with uridine were selected as the IVs (Table 2). Moreover, the *F*-statistic of each IV was calculated as a measure of weak instrumental variable bias, using the following formula: $\mathrm{F}=\frac{{\mathrm{R}}^{2}\times (\mathrm{N}-k-1)}{1-{\mathrm{R}}^{2}}$, where R^2^ indicates the genetic variance in uridine explained by each SNP, N is the exposure dataset sample size, and *k* indicates the number of IVs^22^. IVs with an *F*-statistic less than 10 were considered too weak to represent the exposure and were thus removed^23^. The minor allele frequency of the IVs was not less than 0.01 which supported minor statistical bias due to low confidence.

**Table 2:** Characteristics of SNPs associated with uridine and their associations with AF.

SNP	Chr:pos	EA/OA	EAF	R^2^ (%)	F	Uridine			AF (Nielsen et al*.*)			AF HRC			FinnGen		
						Beta	SE	*p*-value	Beta	SE	*p*-value	Beta	SE	*p*-value	Beta	SE	*p*-value
rs2686796	7:48,102,911	T/C	0.4524	6.993	38.435	0.0094	0.0017	1.89E−08	− 0.0074	0.0067	0.269	− 0.0142	0.0072	0.048	− 0.0285	0.0149	0.055
rs532545	1:20,915,172	T/C	0.3065	6.993	38.435	− 0.0095	0.0017	3.41E−08	0.0158	0.0071	0.026	0.0186	0.0077	0.015	− 0.0385	0.0183	0.035
rs762669	22:50,943,423	A/G	0.2043	6.303	31.198	0.0112	0.0019	1.21E−09	− 0.0171	0.0085	0.044	− 0.0052	0.0096	0.588	− 0.0137	0.0209	0.512

*SNP* single-nucleotide polymorphism, *Chr* chromosome, *pos* position, *EA* effect allele, *OA* other allele, *EAF* effect allele frequency, *R*^*2*^ percentage of the variation of uridine explained by the SNP, *Beta* estimate of the effect of the association, *SE* standard error, *F F* statistic, *AF* atrial fibrillation, *AF HRC* Atrial Fibrillation Haplotype Reference Consortium.

### Statistical analysis

We used the AF datasets from Nielsen et al. in the analysis of the primary outcome because of their large size and populations largely of European descent. The inverse variance weighted (IVW) method combined the Wald ratios of each valid SNP to calculate a pooled estimate, which is widely used in MR studies^24^. The main MR analysis method was the random-effects IVW method, which provides more reliable estimates when heterogeneity is present among SNPs^25^. To verify the robustness of the causal association between plasma uridine and AF, we replicated the IVW analysis using two AF GWAS from the AF HRC study and FinnGen Consortia aforementioned, and performed a meta-analysis to assess the overall situation. Also, a series of supplementary analyses were conducted, including weighted median^26^, simple median and simple mode^27^. Both the fixed-effects and random-effects models were considered for meta-analysis, depending on the heterogeneity among these MR results from different consortia. For meta-analysis, the *p*-value of Cochran’s Q statistic < 0.1 or I^2^ > 50% was deemed to be existing heterogeneity^28,29^.

For MR analysis, the I^2^ statistic and Cochran’s Q were calculated for the IVW model to assess heterogeneity among SNPs. If the *p*-value of Cochran’s Q was less than 0.05 or I^2^ was higher than 25%, we assumed heterogeneity among IVs^30^. In addition, pleiotropic bias was assumed to exist if the MR-Egger intercept’s *p*-value was less than 0.05^31^. To detect highly influential SNPs, we performed leave-one-out analysis^32^. The following formula was used to calculate the proportion of uridine phenotypic variance explained by each IV (R^2^): ${\mathrm{R}}^{2}=\frac{2 \times \mathrm{ EAF }\times (1 -\mathrm{ EAF}) \times {\mathrm{beta}}^{2}}{(2 \times \mathrm{ EAF }\times (1 -\mathrm{ EAF}) \times {\mathrm{beta}}^{2}) + 2 \times \mathrm{ EAF }\times (1 -\mathrm{ EAF}) \times \mathrm{ se }\times \mathrm{ N }\times {\mathrm{beta}}^{2})}$. Here, the terms “EAF”, “beta”, “se”, and “N” stand for, respectively, “effect allele frequency”, “effect size”, “standard error”, and “sample size”^30^.

In light of the fact that this study involves multiple analyses, associations at a *p*-value (*p* < 0.017) after Bonferroni correction were deemed significant. We also calculated the power, analysis to determine the sample size required to assess the outcome measure, percentage of cases, sum of R^2^, and took 0.05 as the type I error rate in the online tool mRnd^33^. The MR analyses were conducted using the R software 4.2.2^34^ with the TwoSampleMR package. And the meta package was used to perform meta-analysis.

## Results

According to the results obtained by MR Steiger filtering (Table S3), three SNPs selected as the IVs explained more of the variance in plasma uridine levels than in AF, indicating that there was no reverse causality between the exposure and outcome variables. Together, these SNPs explained 20.3% of the phenotypic variability in plasma uridine (Table 2). The three SNPs had *F*-statistics higher than 30, which suggests there was a minor weak IVs bias in this study. In addition, the data sources for plasma uridine and AF have no sample overlap.

**Figure 2:**
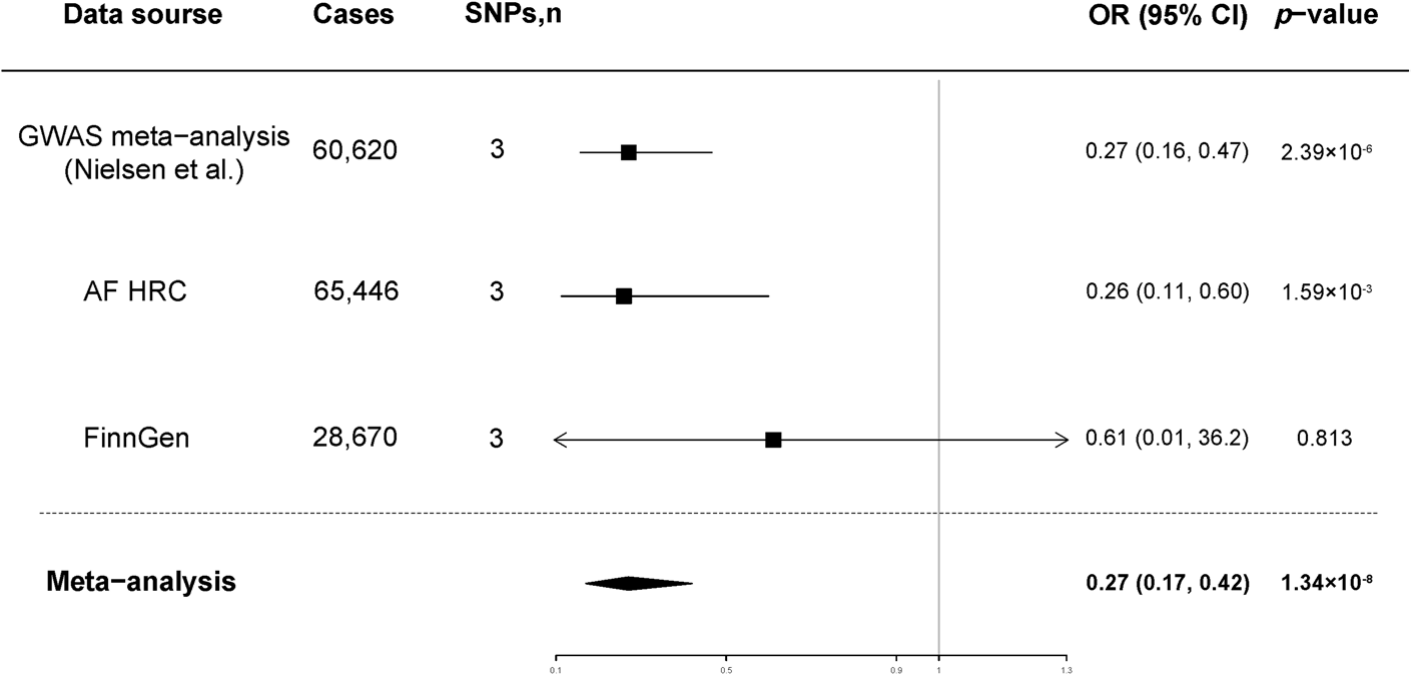
Association between genetically proxied plasma uridine level and the risk of AF. *AF HRC* Atrial Fibrillation Haplotype Reference Consortium, *GWAS* genome-wide association study, *AF* atrial fibrillation, *OR* odds ratio, *CI* confidence interval.

**Figure 3:**
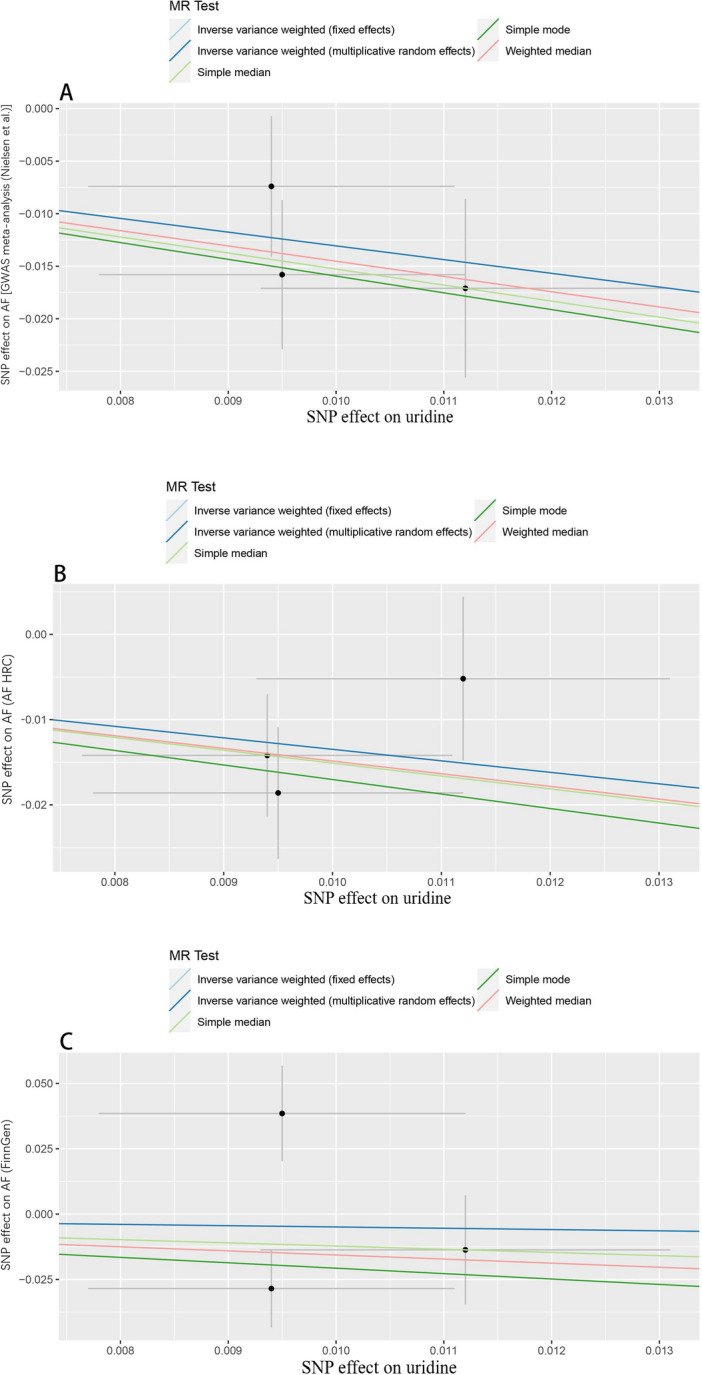
Scatter plot of five MR methods for assessing the association of the plasma uridine level with the risk of AF based on GWAS meta-analysis (Nielsen et al.) (**A**), AF HRC (**B**) and FinnGen (**C**). *AF HRC* Atrial Fibrillation Haplotype Reference Consortium, *GWAS* genome-wide association study, *AF* atrial fibrillation.

For the primary analysis, at an odds ratio (OR) of 0.27, the statistical power was 100% for detecting significant differences with a sample size of 1,030,836 (60,620 cases). According to the standard IVW analysis, higher genetically instrumented plasma uridine levels were significantly associated with a lower tendency to suffer from AF in both GWAS meta-analyses at a statistically significant level after Bonferroni correction (*p* < 0.017): (1) in the AF GWAS datasets conducted by Nielsen et al. (OR 0.27; 95% confidence interval [CI] 0.16, 0.47; *p* = 2.39 × 10^–6^) and (2) in the AF HRC (OR 0.26; 95% CI 0.11, 0.60; *p* = 1.59 × 10^–3^). Additionally, for the FinnGen Consortia, the MR estimate was positive, albeit nonsignificant and of relatively low magnitude (Figs. 2, 3). We included the three outcome datasets in a meta-analysis (OR 0.27; 95% CI 0.17, 0.42; *p* = 1.34 × 10^–8^), which demonstrated the reliability of results (Fig. 2). Several complementary statistical analyses with various assumptions were conducted to further support the primary results (Table 3). And the MR effect size for each SNP on AF was shown in the forest plot (Supplemental Fig. 1).

**Table 3:** Estimates of the causal effect of plasma uridine level on AF by four complementary methods.

Data source	Method	OR	95% CI	*p*-value
GWAS meta-analysis (Nielsen et al*.*)	IVW (fixed-effects)	0.27	(0.12, 0.62)	2.20 × 10^–03^
	Weighted median	0.23	(0.08, 0.65)	5.22 × 10^–03^
	Simple median	0.22	(0.07, 0.64)	5.83 × 10^–03^
	Simple mode	0.20	(0.06, 0.65)	0.115
AF HRC	IVW (fixed-effects)	0.26	(0.10, 0.65)	3.87 × 10^–03^
	Weighted median	0.23	(0.07, 0.77)	0.018
	Simple median	0.22	(0.06, 0.75)	0.016
	Simple mode	0.18	(0.04, 0.91)	0.173
FinnGen	IVW (fixed-effects)	0.61	(0.08, 4.52)	0.629
	Weighted median	0.21	(0.01, 3.50)	0.276
	Simple median	0.29	(0.01, 6.37)	0.435
	Simple mode	0.13	(0.005, 3.4)	0.343

*AF HRC* Atrial Fibrillation Haplotype Reference Consortium, *SNPs* single-nucleotide polymorphisms, *IVW* inverse-variance weighted, *OR* odds ratio, *CI* confidence interval.

**Figure 4:**
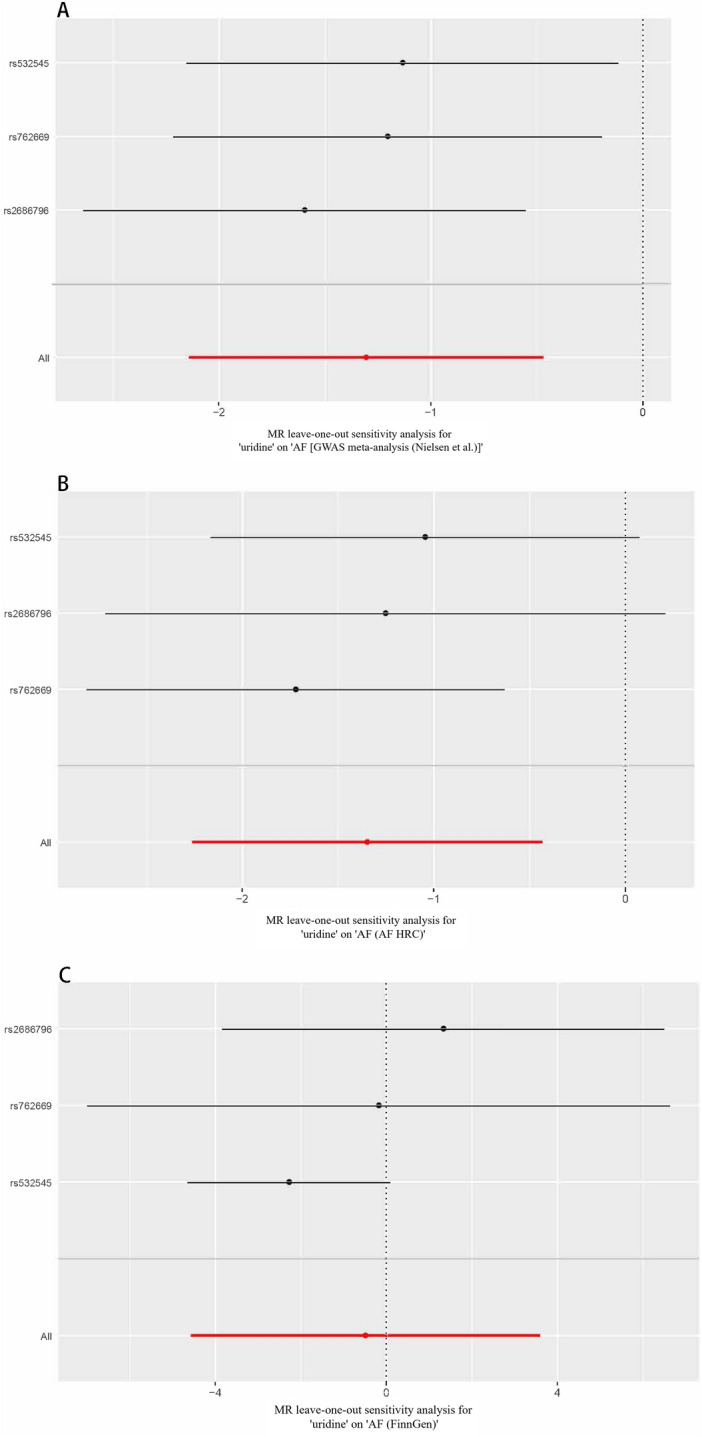
MR leave-one-out analysis of the association of plasma uridine level with the risk of AF based on GWAS meta-analysis (Nielsen et al.) (**A**), AF HRC (**B**) and FinnGen (**C**). *AF HRC* Atrial Fibrillation Haplotype Reference Consortium, *GWAS* genome-wide association study, *AF* atrial fibrillation.

There was no evidence of heterogeneity or horizontal pleiotropy among the IVs, the *p*-values for Cochran’s Q and MR-Egger Intercept were much higher than 0.017 in both GWAS meta-analyses (Table 4). However, the I^2^ statistic calculated based on the FinnGen Consortium was 76%, indicating high heterogeneity among the SNPs. Therefore, we employed the random-effect IVW as the primary statistical approach to evaluate the causal relationship between plasma uridine level and the risk of AF. No single SNP significantly impacted the results in a leave-one-out analysis (Fig. 4).

**Table 4:** Evaluation of pleiotropy and heterogeneity.

Data source	Heterogeneity		Pleiotropy	
	I^2^ (%)	Cochran’s Q *p*	MR-Egger intercept	MR-Egger intercept *p*
GWAS meta-analysis (Nielsen et al.)	0	0.656	0.02	0.757
AF HRC	0	0.433	− 0.07	0.441
FinnGen	76	0.017	0.04	0.934

*AF HRC* Atrial Fibrillation Haplotype Reference Consortium, *MR-Egger* Mendelian randomisation-Egger.

For meta-analysis, the *p*-value for Cochran’s Q was 0.922 and the I^2^ was 0%, indicating no heterogeneity among the MR results. The fixed-effects meta-analysis method is reliable in our studies.

## Discussion

In this MR study, we first explored the causal association between plasma uridine levels and AF using publicly available GWAS datasets. We found evidence from a genetic perspective that an increased plasma uridine level reduces the risk of AF. Furthermore, we duplicated the results using a series of complementary MR methods. According to our analysis of the FinnGen Consortium dataset, there was high heterogeneity among IVs and an association of uridine with AF were directionally coherent (albeit lacking statistical significance). As the primary analysis approach, we applied the IVW method in the multiplicative random-effects model, which enables reliable estimation of heterogeneity among SNPs. Meta-analyses of the three datasets combined allowed us to obtain an integrated MR estimate, which facilitated causal inference. Our findings will aid the development of the novel preventive strategies for AF.

Uridine, a pyrimidine nucleoside, is more abundant in human plasma than other nucleosides^35^ and is critical in the synthesis of endogenous pyrimidine, glycogen, biomembranes and the like^36^. Uridine modulates heart rhythm disorders through its participation in myocardial glycogen resynthesis and ATP production for ion transport systems under pathological conditions^37^. Fibrosis has been well established and exerts a crucial effect in the development of AF^38^. Specifically, mitochondrial reactive oxygen species (ROS) are activated under various pathological and stress conditions, in turn activate p38 and ERK1/2 to accelerate the transcription of fibrotic genes^39^. Liu et al*.* found that uridine supplementation promoted tissue recovery in cardiac injury models, and decreased fibrosis and inflammatory cytokine levels^3^. Oxidative stress and inflammation play crucial roles in the onset and development of AF^38^. Additionally, uridine treatment can significantly decrease the levels of oxidative stress and inflammation in vitro^40^. The positive effects of uridine in reducing oxidative stress and inflammation might be involved with the activation of the mitoK_ATP_ channel^40,41^. And thus, it preserves the structure and function of mitochondria. Thereby ultimately ameliorating fibrosis in the myocardium.

Our findings coincide with a prospective cohort study based on the Atherosclerosis Risk in Communities study, which showed that a higher plasma uridine level was associated with a decreased risk of AF (hazard ratio 0.85; 95% CI 0.79, 0.92; *p* = 1.3 × 10^–4^)^42^. In addition, an observational cohort study based on the Framingham Heart Study yielded a similar, albeit nonsignificant, result regarding uridine as a protective factor for AF (hazard ratio 0.84; 95% CI 0.70, 1.00; *p* = 0.052)^43^. Observational studies can be influenced by potential confounders, which makes reverse causation difficult to determine and distorts true causal associations. To figure out whether uridine reduces the risk of AF, further prospective clinical trials based on large populations may shed light on this but expend a relative amount of time. This MR analysis based on datasets from GWASs of mountains of European population sought to demonstrate this issue. A recent MR investigation proposed similar conclusions with us. Their independent study extracted different IVs with a significant threshold at *p* < 1 × 10^–5^ and came to similar results^44^.

A strength of this study was its use of the MR analysis framework, which can reliably estimate causal associations. Also, our MR estimates strengthened the reliability of the casual inference between plasma uridine levels and AF with low confounding bias. Additionally, no horizontal pleiotropy was discovered for any of the outcomes examined using MR-Egger regression (a sensitivity analysis), MR Steiger filtering and PhenoScanner V2. There is thus a low probability of pleiotropic effects in this investigation. Our findings provide a foundation for further research on the prophylactic potential against AF of increasing plasma uridine levels.

However, this study had certain limitations worth recognizing. First, the quantity of uridine-associated SNPs was too few for performing the MR Pleiotropy Residual Sum and Outlier method, which were deemed a global test to access pleiotropy among IVs and provides an MR estimate after removing pleiotropic outliers^45^. It is still worth noting that a potential pleiotropy bias might have been present in this study. Second, the I^2^ statistic calculated based on the FinnGen Consortium was 76%, suggesting high heterogeneity among the three SNPs. This might be due to the amount of selected IVs was too few, larger GWAS datasets of uridine are needed to detect more uridine-associated SNPs. Third, given that this study was based on summary-level data, potential nonlinear associations between plasma uridine and AF were not examined. Fourth, the MR estimates based on the FinnGen Consortium database were significantly different from the others, which could be attributed to the fact that the AF cases in that database include some atrial flutter patients. Moreover, the AF HRC collaboration includes individuals of non-European ancestry (almost 9%), thus the demographic stratification may increase bias. Owing to this MR study was limited to participants who were mostly of European heritage, it is difficult to generalize to populations of other descents. Investigating the causal relationships among various populations would be of great interest. Future research is necessary to expand on our findings.

## Conclusions

This MR study provided evidence from a genetic perspective suggesting that a higher plasma uridine level can reduce the propensity to suffer from AF. These results might open up new vistas for prevention strategies in AF.

### Supplementary Information

Supplementary Information.

## Data Availability

This study relied entirely on data from publicly available databases. The original data reported in the study are available in the article and Supplementary Materials. Inquiries should be addressed to the corresponding author.

## Abbreviations

AF: Atrial fibrillation
AF HRC: Atrial Fibrillation Haplotype Reference Consortium
CI: Confidence interval
EAF: Effect allele frequency
GWAS: Genome-wide association study
ICD: International Classification of Diseases
IVs: Instrumental variables
IVW: Inverse-variance weighted
KORA: Cooperative Health Research in the Region of Augsburg
LD: Linkage disequilibrium
MR: Mendelian randomisation
OR: Odds ratio
RCT: Randomised controlled trials
ROS: Reactive oxygen species
se: Standard error
SNPs: Single nucleotide polymorphisms
STROBE-MR: Strengthening the reporting of observational studies in epidemiology using Mendelian randomisation
TwinsUK: UK Adult Twin Registry
