# Supplementary material for: Protective effect of uridine on atrial fibrillation: a Mendelian randomisation study
Source: Sci Rep. 2023 Nov 10;13:19639. doi: 10.1038/s41598-023-47025-8 (PMC10638443; doi:10.1038/s41598-023-47025-8)
Supplement: Supplementary file 1 — Supplementary Information. [file 41598_2023_47025_MOESM1_ESM.pdf]

Protective Effect of Uridine on Atrial Fibrillation: A Mendelian Randomisation Study

Xintian Xu[1,2], Xiaoyu Zhang[1,2], Shiyao Cheng[1,2], Qinglang Li[1,2], Cai Chen[1,2] and Mao Ouyang[1,2]

[1]Department of Cardiology, The Sixth Affiliated Hospital, Sun Yat-sen University, Guangzhou, People’s Republic of China.

[2]Biomedical Innovation Center, The Sixth Affiliated Hospital, Sun Yat-sen University

Correspondence: Mao Ouyang, ouym7@mail.sysu.edu.cn, Department of Cardiology, The Sixth Affiliated Hospital, Sun Yat-sen University, 26 Yuancun Erheng Road, Guangzhou, Guangdong, 510655, People's Republic of China.

Supplementary Material

Supplementary Table 1. STROBE-MR Checklist. (Skrivankova et al., 2021)

Item No.	Section	Checklist item	Manuscript section and paragraph
**Title and abstract**			
1	Title and abstract	Indicate mendelian randomization (MR) as the study's design in the title and/or the abstract if that is a main purpose of the study.	Title and abstract
**Introduction**			
2	Background	Explain the scientific background and rationale for the reported study. What is the exposure? Is a potential causal relationship between exposure and outcome plausible? Justify why MR is a helpful method to address the study question.	Introduction, paragraphs 1-2
3	Objectives	State specific objectives clearly, including prespecified causal hypotheses (if any). State that MR is a method that, under specific assumptions, intends to estimate causal effects.	Introduction, paragraph 3
**Methods**			
4	Study design and data sources	Present key elements of the study design early in the article. Consider including a table listing sources of data for all phases of the study. For each data source contributing to the analysis, describe the following:	Methods, "Study design" section; Figure 1
	a	Setting: Describe the study design and the underlying population, if possible. Describe the setting, locations, and relevant dates, including periods of recruitment, exposure, follow-up, and data collection, when available.	Methods, "Study design" section
	b	Participants: Report the eligibility criteria and the sources and methods of selection of participants. Report the sample size and whether any power or sample size calculations were carried out prior to the main analysis.	Methods, "Data Sources and SNPs selection" and "Statistical analysis" sections; Table 1;

	c	Describe measurement, quality control, and selection of genetic variants.	Methods, "Data Sources and SNPs selection" section
	d	For each exposure, outcome, and other relevant variables, describe methods of assessment and diagnostic criteria for diseases.	Methods, "Data Sources and SNPs selection" section
	e	Provide details of ethics committee approval and participant informed consent, if relevant.	Methods, "Study design" section
5	Assumptions	Explicitly state the 3 core instrumental variable (IV) assumptions for the main analysis (relevance, independence, and exclusion restriction), as well assumptions for any additional or sensitivity analysis.	Methods, "Study design" and "Statistical analysis" sections; Figure 1
6	Statistical methods: main analysis	Describe statistical methods and statistics used.	Methods, "Statistical analysis" sections
	a	Describe how quantitative variables were handled in the analyses (ie, scale, units, model).	Methods, "Statistical analysis" sections
	b	Describe how genetic variants were handled in the analyses and, if applicable, how their weights were selected.	Methods, "Statistical analysis" sections
	c	Describe the MR estimator (eg, 2-stage least squares, Wald ratio) and related statistics. Detail the included covariates and, in case of 2-sample MR, whether the same covariate set was used for adjustment in the 2 samples.	Methods, "Statistical analysis" sections
	d	Explain how missing data were addressed.	N/A
	e	If applicable, indicate how multiple testing was addressed.	Method, "Statistical analysis" sections
7	Assessment of assumptions	Describe any methods or prior knowledge used to assess the assumptions or justify their validity.	Methods, "Statistical analysis" sections

8	Sensitivity analyses and additional analyses	Describe any sensitivity analyses or additional analyses performed (eg, comparison of effect estimates from different approaches, independent replication, bias analytic techniques, validation of instruments, simulations).	Methods, "Statistical analysis" sections
9	Software and preregistration		
	a	Name statistical software and package(s), including version and settings used.	Methods, "Statistical analysis" sections
	b	State whether the study protocol and details were preregistered (as well as when and where).	N/A
**Results**			
10	Descriptive data		
	a	Report the numbers of individuals at each stage of included studies and reasons for exclusion. Consider use of a flow diagram.	Table 1
	b	Report summary statistics for phenotypic exposure(s), outcome(s), and other relevant variables (eg, means, SDs, proportions).	Table 1
	c	If the data sources include meta-analyses of previous studies, provide the assessments of heterogeneity across these studies.	Results, paragraph 4
	d	For 2-sample MR:<br>i. Provide justification of the similarity of the genetic variant–exposure associations between the exposure and outcome samples.<br>ii. Provide information on the number of individuals who overlap between the exposure and outcome studies.	Results, paragraph 1; Supplementary Table 2
11	Main results		
	a	Report the associations between genetic variant and exposure and between genetic variant and outcome, preferably on an interpretable scale.	Table 2

	b	Report MR estimates of the relationship between exposure and outcome and the measures of uncertainty from the MR analysis, on an interpretable scale, such as odds ratio or relative risk per SD difference.	Results, paragraph 2
	c	If relevant, consider translating estimates of relative risk into absolute risk for a meaningful time period.	N/A
	d	Consider plots to visualize results (eg, forest plot, scatterplot of associations between genetic variants and outcome vs between genetic variants and exposure).	Figure 2 and Figure 3
12	Assessment of assumptions		
	a	Report the assessment of the validity of the assumptions.	Results, paragraph 3
	b	Report any additional statistics (eg, assessments of heterogeneity across genetic variants, such as I2, Q statistic, or E-value).	Results, paragraph 3
13	Sensitivity analyses and additional analyses		
	a	Report any sensitivity analyses to assess the robustness of the main results to violations of the assumptions.	Results, paragraph 3
	b	Report results from other sensitivity analyses or additional analyses.	Results, paragraph 3
	c	Report any assessment of the direction of the causal relationship (eg, bidirectional MR).	Results, paragraph 1
	d	When relevant, report and compare with estimates from non-MR analyses.	N/A
	e	Consider additional plots to visualize results (eg, leave-one-out analyses).	Figure 4
**Discussion**			
14	Key results	Summarize key results with reference to study objectives.	Discussion, paragraph 1
15	Limitations	Discuss limitations of the study, taking into account the validity of the IV assumptions, other sources of potential bias, and imprecision. Discuss both direction and magnitude of any potential bias and any efforts to address them.	Discussion, paragraph 5

16	Interpretation		
	a	Meaning: Give a cautious overall interpretation of results in the context of their limitations and in comparison with other studies.	Discussion, paragraph 2 and 3
	b	Mechanism: Discuss underlying biological mechanisms that could drive a potential causal relationship between the investigated exposure and the outcome, and whether the gene-environment equivalence assumption is reasonable. Use causal language carefully, clarifying that IV estimates may provide causal effects only under certain assumptions.	Discussion, paragraph 3 and 4
	c	Clinical relevance: Discuss whether the results have clinical or public policy relevance, and to what extent they inform effect sizes of possible interventions.	Conclusions
17	Generalizability	Discuss the generalizability of the study results (a) to other populations, (b) across other exposure periods/timings, and (c) across other levels of exposure.	Discussion, paragraph 5
**Other Information**			
18	Funding	Describe sources of funding and the role of funders in the present study and, if applicable, sources of funding for the databases and original study or studies on which the present study is based.	Funding statement section
19	Data and data sharing	Provide the data used to perform all analyses or report where and how the data can be accessed, and reference these sources in the article. Provide the statistical code needed to reproduce the results in the article or report whether the code is publicly accessible and, if so, where.	Data availability statement section
20	Conflicts of interest	All authors should declare all potential conflicts of interest.	Conflicts of interest section

Reference:

Skrivankova, V.W., Richmond, R.C., Woolf, B.A.R., Yarmolinsky, J., Davies, N.M., Swanson, S.A., et al. (2021). Strengthening the Reporting of Observational Studies in Epidemiology Using Mendelian Randomization: The STROBE-MR Statement. JAMA 326, 1614-1621. doi: 10.1001/jama.2021.18236

Supplementary Table 2. Contributing studies of the datasets used for analysis.

Use in this MR	Phenotype	Data source	Contributing studies	Sample overlap
Exposure	uridine	Metabolomics GWAS server	KORA, TwinsUK.	/
Outcomes	Atrial fibrillation	Nielsen et al.	HUNT, deCODE, MGI, DiscovEHR, UK Biobank, AFGen Consortium.	0
		AF HRC	AFGen consortium, Broad AF study, UK Biobank, Biobank Japan, AFCT, AFLMU, AGES, ANGES, ARIC, Australian Familial AF Study, BEAT-AF, Biobank Japan, BioMe, BioVU, Broad AF Study, CCAF, CHS, Corogene, Danish AF Study, Duke, EAST AFNET4, EGCUT 370, EGCUT Omni, FHS, FINCAVAS, GENAF, German Heart Center Controls, GerMIFS, GGAF, GRADE, GS:SFHS, Hopkins, HVH, Incor Warfarin Study, Intermountain, LURIC, MDCS, MESA, MGH AF, MGH CAMP, MGH DOFEGEN, MGH Stroke, MPP AF, MPP Echo, Penn, PHB (MEG), PHB (MEGA Ex), PHB (MEGA), PREVEND, PROSPER, RS1, RS2, RS3, SHIP, SiGN Group1, SiGN Group2, SiGN Group4, SiGN Group5, SiGN Group6, SiGN Group7, SiGN Group8, SiGN Group9, SPHFC, TCAI, TWINGENE, UCSF, UMass, VAFAR, Vanderbilt AF Registry, WGHS, WTCCC2 Munich.	0
		FinnGen	FinnGen.	0

KORA, Cooperative Health Research in the Region of Augsburg; TwinsUK, The UK Adult Twin Registry; HUNT, The Nord-Trøndelag Health Study; MGI, the Michigan Genomics Initiative; AF HRC, Atrial Fibrillation Haplotype Reference Consortium.

Supplementary Table 3. Results of MR Steiger direction test.

Data source	Outcome	SNP	rsq.exposure	rsq.outcome	steiger_dir*	steiger_pval
GWAS meta-analysis (Nielsen et al.)	AF	rs2686796	3.89E-03	1.18E-06	TRUE	6.35E-08
		rs532545	3.98E-03	4.80E-06	TRUE	7.87E-08
		rs762669	4.42E-03	3.93E-06	TRUE	1.25E-08
AF HRC	AF	rs2686796	3.89E-03	6.61E-06	TRUE	1.42E-07
		rs532545	3.98E-03	9.92E-06	TRUE	1.36E-07
		rs762669	4.42E-03	4.99E-07	TRUE	7.07E-09
FinnGen	AF	rs2686796	3.89E-03	2.24E-05	TRUE	6.04E-07
		rs532545	3.98E-03	2.69E-05	TRUE	5.52E-07
		rs762669	4.42E-03	2.61E-06	TRUE	1.96E-08

*SNPs with "TRUE" MR Steiger results suggest causality in the expected direction (ie, thoses explaining more variance in exposure than in outcome) and will not be removed from the study.

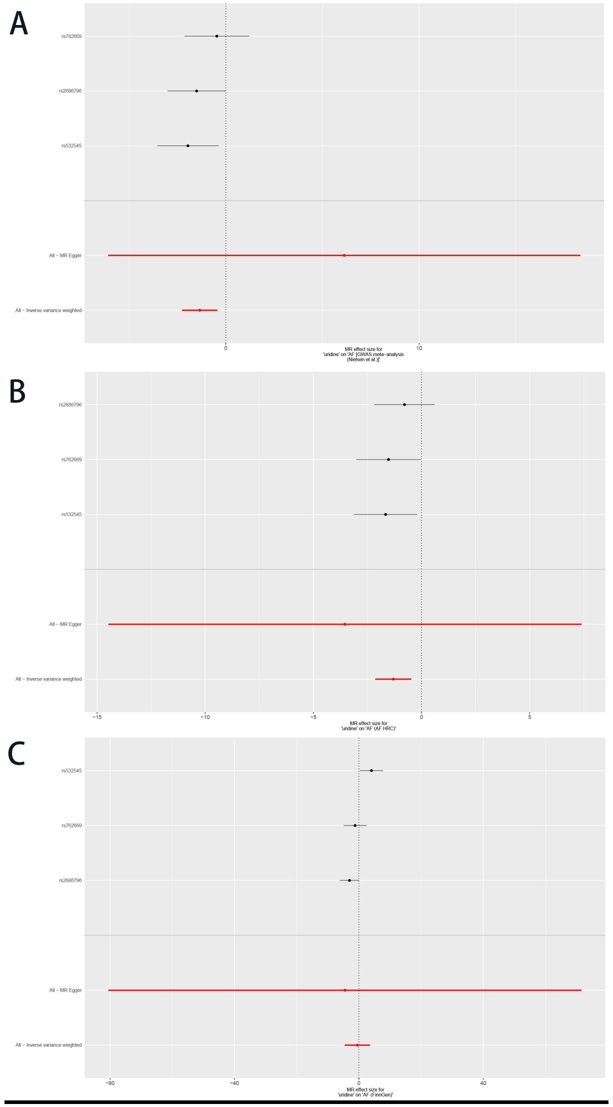

Supplementary Figure 1. The forest plot of MR analysis for 3 SNPs with the risk of AF based on GWAS meta-analysis (Nielsen et al.) (A), AF HRC (B) and FinnGen (C).

AF HRC, Atrial Fibrillation Haplotype Reference Consortium; GWAS, genome-wide association study; AF, atrial fibrillation
